# Establishment and Validation of Prognostic Nomograms for Patients With Parotid Gland Adenocarcinoma Not Otherwise Specified: A SEER Analysis From 2004 to 2016

**DOI:** 10.3389/fsurg.2021.799452

**Published:** 2022-01-11

**Authors:** Zi-Meng Wang, Zuo-Lin Xiang

**Affiliations:** Department of Radiation Oncology, Shanghai East Hospital, School of Medicine, Tongji University, Shanghai, China

**Keywords:** parotid gland, adenocarcinoma not otherwise specified, SEER database, prognosis, nomogram

## Abstract

**Background:** Parotid gland adenocarcinoma not otherwise specified (PANOS) is a rare malignant tumor with limited data on its characteristics and prognosis. This research is aimed at characterizing PANOS and developing prognostic prediction models for patients with PANOS.

**Methods:** Cases from 2004–2016 were selected from the Surveillance, Epidemiology, and End Results (SEER) Program database. Univariate and multivariate Cox regression were applied to ascertain the factors associated with survival. Competing risk analysis and Gray's tests were employed to analyze cancer-specific death. Propensity score matching (1:1) was conducted to reduce the influence of confounding variables.

**Results:** A total of 446 patients with a median age of 66 years were selected, of which 307 were diagnosed with stage III/IV PANOS. The 5-year overall survival (OS) rate of all patients was 51.8%, and the median survival time was 66 months. Surgical treatment clearly improved survival time (*p* < 0.001). In the subgroup analysis, radiotherapy showed survival benefits in patients with stage III/IV disease (*p* < 0.001). Multivariate Cox regression analyses showed that age, T classification, N classification, M classification and surgery were independent prognostic indicators for OS; T classification, N classification, M classification and surgery were independent risk factors for cancer-specific survival (CSS). In addition, age was independently associated with other cause-specific death. Based on the results of multivariate analysis, two nomograms were developed and verified by the concordance index (C-index) (0.747 and 0.780 for OS and CSS) and the area under the time-dependent receiver operating characteristic (ROC) curve (0.756, 0.764, and 0.819 regarding for nomograms predicting 3-, 5-, and 10- year OS, respectively and 0.794, 0.789, and 0.806 for CSS, respectively).

**Conclusions:** Our study clearly presents the clinicopathological features and survival analysis of patients with PANOS. In addition, our constructed nomogram prediction models may assist physicians in evaluating the individualized prognosis and deciding on treatment for patients.

## Introduction

As a rare malignant tumor, parotid gland carcinoma accounts for 1–3% of all head and neck malignancies (1). Most head and neck malignancies are squamous cell carcinoma, but parotid gland carcinomas are more diverse, with 24 different histologic subtypes according to the 2017 World Health Organization (WHO) classification (2, 3). The most common pathological types of parotid gland carcinomas are mucoepidermoid carcinoma and adenoid cystic carcinoma, which has been systematically studied (4–6). Adenocarcinoma not otherwise specified (ANOS) is also a major subtype (7–10) which refers to carcinoma that has different levels of glandular differentiation in histology but cannot be attributed to a specific type. Its incidence ranges from the second to the fourth in parotid gland carcinoma based on different retrospective studies (7, 8, 11–13). Although a few case series (14, 15) have shown that the biological behavior of parotid gland adenocarcinoma not otherwise specified (PANOS) is highly malignant, large sample research regarding to its clinical features and long-time survival is still lacking due to the paucity of patients.

In order to fully evaluate the clinicopathological characteristics and prognosis of patients with PANOS, we extracted cases from the SEER database between 2004 and 2016 and conducted a comprehensive analysis. Meanwhile, we constructed two nomograms to help physicians predict the overall survival (OS) and cancer-specific survival (CSS) of these patients directly.

## Methods

### Patient Selection

**Figure 1:**
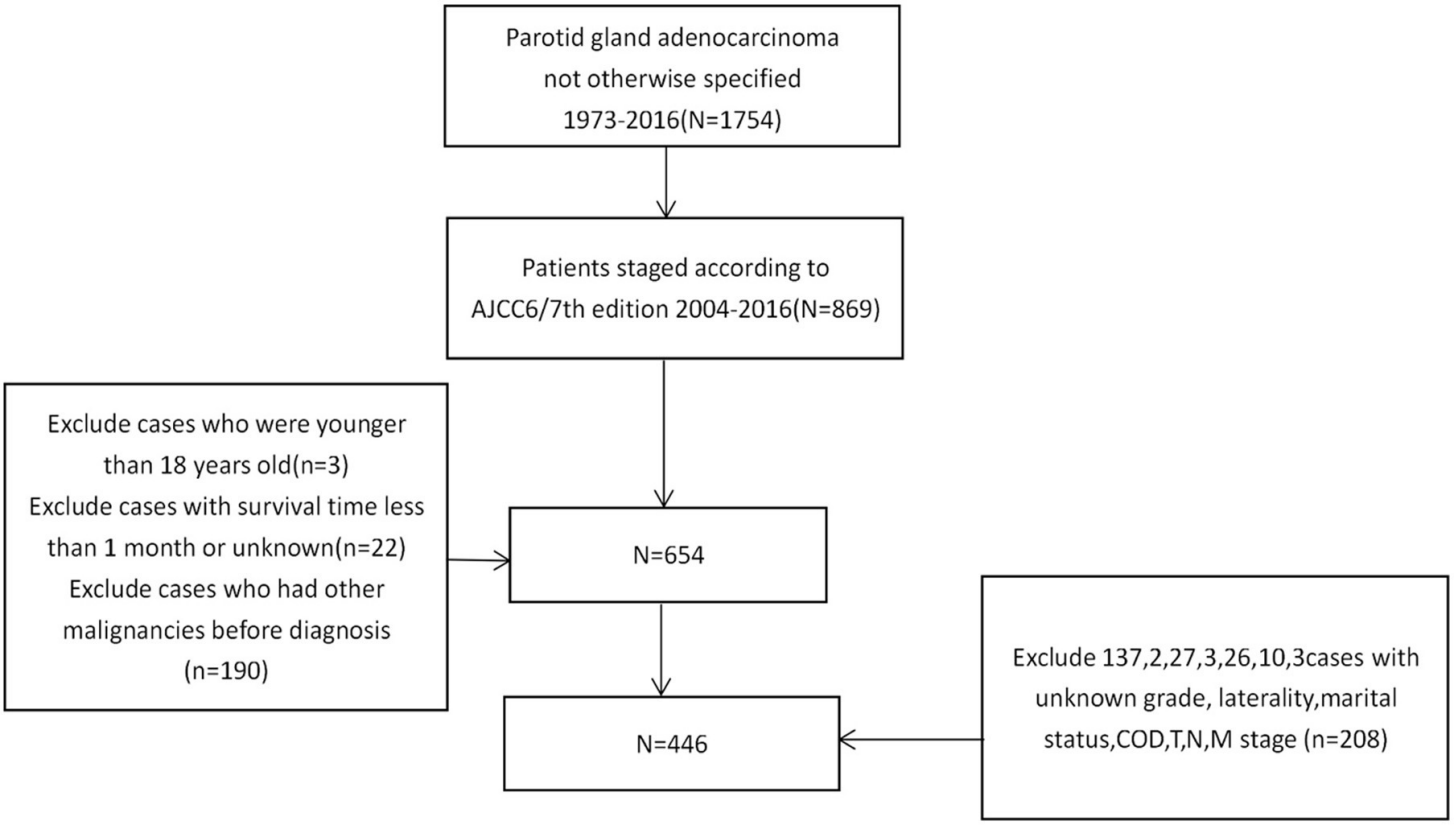
The flow diagram of the selection process for the study cohort.

We used SEER^*^Stat software (version 8.3.9) to download data from the SEER^*^Stat Database. Cases were extracted using keywords: (1) primary site-labeled = C07.9; (2) ICDO-3 Hist/behav, malignant: 8140/3. Specific information regarding age, gender, race, laterality, pathological grade, radiotherapy, chemotherapy, surgery, and the AJCC 6/7th TNM stage was downloaded. The exclusion criteria were as follows: (1) patients without complete AJCC 6/7th TNM stage data; (2) patients had a survival time of 1 month or less; (3) patients were younger than 18 years old; (4) Not the patient's first tumor; (5) patients without information regarding race, laterality, grade, T classification, N classification and M classification (Figure 1).

### Statistical Analysis

The primary endpoint was overall survival and the secondary endpoint was cancer-specific survival. Univariate Cox regression analyses were performed to filter out significant variables related to OS. Kaplan-Meier curves were plotted and compared using the log-rank test for each significant variable. Competing risk analysis and Gray's tests were utilized to test the differences in cancer-specific death between subgroups. The cumulative incidence function curves were also depicted. Multivariate Cox regression analyses were subsequently conducted.

One-to-one propensity score matching (PSM) was performed using nearest neighbor matching method to construct a matched cohort including pairs of radiotherapy and non-radiotherapy subjects. The caliper value was 0.02. Age, gender, race, laterality, marital status, pathological grade, T, N and M stages, stage, surgery and chemotherapy were covariates used for matching.

Nomograms were constructed based on the results of multivariate analyses. Calibration curves were delineated using the Kaplan-Meier method with bootstrap to evaluate the agreement between the nomogram predicted survival rate and the actual survival rate. By calculating the performance of the constructed nomogram, the time-dependent receiver operating characteristic (ROC) curves were investigated. The C-index and the area under the ROC curve (AUC) were employed to examine the accuracy of the nomogram.

R software (version 4.0.5) was used to conduct all statistical analyses and *p* value < 0.05 was considered statistically significant.

## Results

### Patient Characteristics

Four Hundred and Forty Six PANOS Patients (2004–2016) Were Chosen From the SEER Database (Table 1). Among the 446 Selected Patients, There Were 289 men and 157 Women, With a Male-to-Female Ratio of 1.84. Almost two-Thirds of the Patients (307/446) Had III/IV Stage Tumors. The Results Show That There Was a Tendency to Poorly Differentiated Tumors, Followed by Moderately Differentiated and Undifferentiated Tumors. And Also, in Selected Patients, Nearly Half (50.9%) Had Nodal Involvement While Only 9.6% Had Distant Metastasis. Surgery Was the Main Treatment Modality (409/446). 91.70% of Patients (319/446) Received Radiotherapy While 22.87% (102/446) Received Chemotherapy.

**Table 1:** Demographic and clinicopathological characteristics of patients and univariate Cox regression analyses of overall survival.

**Characteristics**	**N (%)**	***p-*value**	**HR**	**95% CI**
Age (year)		**<0.001**		
>66	209 (46.9)	Ref	1	
≤ 66	237 (53.1)		0.514	0.389–0.678
Gender		**0.002**		
Female	157 (35.2)	Ref	1	
Male	289 (64.8)		1.594	1.179–2.154
Race		0.958		
White	371 (83.2)	Ref	1	
Black	44 (9.9)	0.673	0.833	0.356–1.947
Other	31 (6.9)	0.738	0.915	0.545–1.538
Laterality		0.616		
Left	199 (44.6)	Ref	1	
Right	247 (55.4)		0.933	0.709–1.228
Marital status		0.349		
Divorced/other	165 (37.0)	Ref	1	
Married	281 (63.0)		0.866	0.654–1.146
Grade		**<0.001**		
G1	25 (5.6)	Ref	1	
G2	122 (27.4)	0.065	3.002	0.933–9.662
G3	221 (49.6)	<0.01	6.165	1.961–19.384
G4	78 (17.4)	0.017	4.204	1.293–13.671
T classification		**<0.001**		
T1	100 (22.4)	Ref	1	
T2	119 (26.7)	<0.01	2.097	1.248–3.523
T3	88 (19.7)	<0.001	3.595	2.134–6.058
T4	139 (31.2)	<0.001	5.621	3.495–9.042
N classification		**<0.001**		
N0	219 (49.1)	Ref	1	
N1	79 (17.7)	<0.001	2.156	1.481–3.138
N2	142 (31.8)	<0.001	3.077	2.232–4.243
N3	6 (1.4)	0.001	3.883	1.686–8.941
M classification		**<0.001**		
M0	403 (90.4)	Ref	1	
M1	43 (9.6)		3.808	2.549–5.689
Stage		**<0.001**		
I	77 (17.3)	Ref	1	
II	62 (13.9)	0.893	1.052	0.501–2.212
III	69 (15.5)	<0.01	2.664	1.432–4.957
IV	238 (53.3)	<0.001	5.720	3.349–9.768
Surgery		**<0.001**		
No	37 (8.3)	Ref	1	
Yes	409 (91.7)		0.214	0.138–0.330
Radiotherapy		0.278		
No	127 (28.5)	Ref	1	
Yes	319 (71.5)		0.838	0.622–1.129
Chemotherapy		**<0.001**		
No	344 (77.1)	Ref	1	
Yes	102 (22.9)		1.771	1.294–2.424

*Bold value means the p-value of a statistically significant factor*.

### Survival Analysis

**Figure 2:**
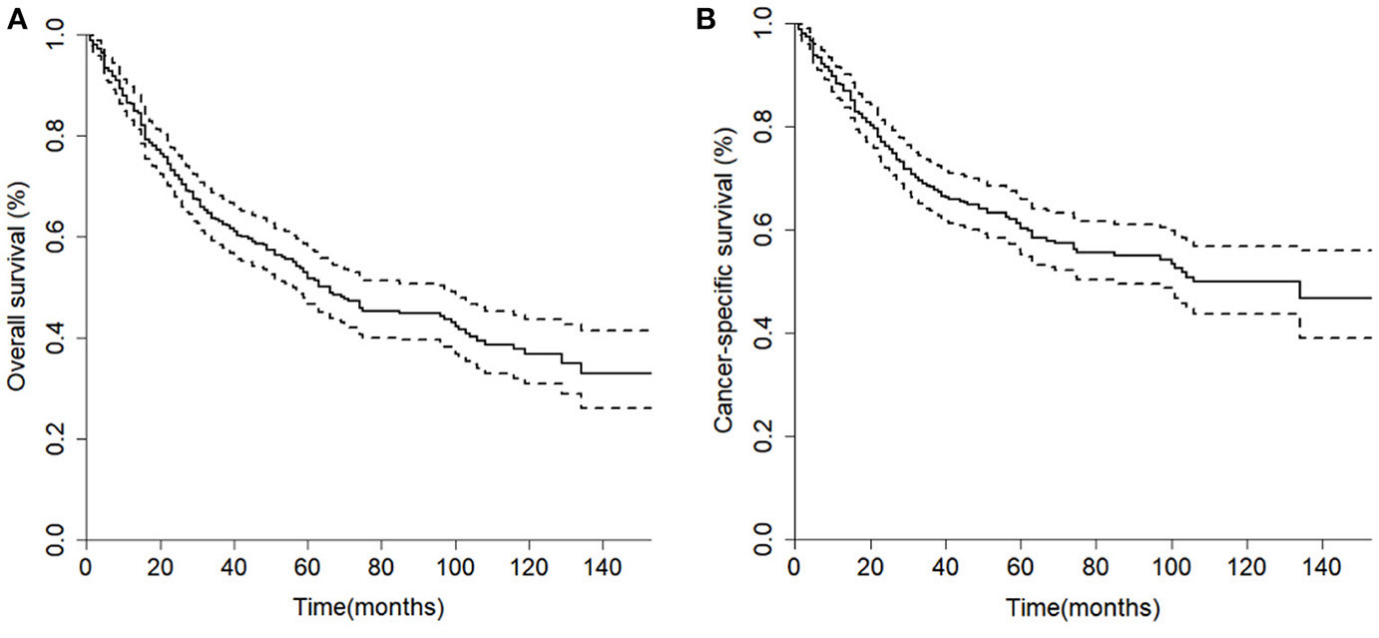
Kaplan-Meier curves of the 446 patients in the cohort. **(A)** Overall survival; **(B)** Cancer-specific survival.

**Figure 3:**
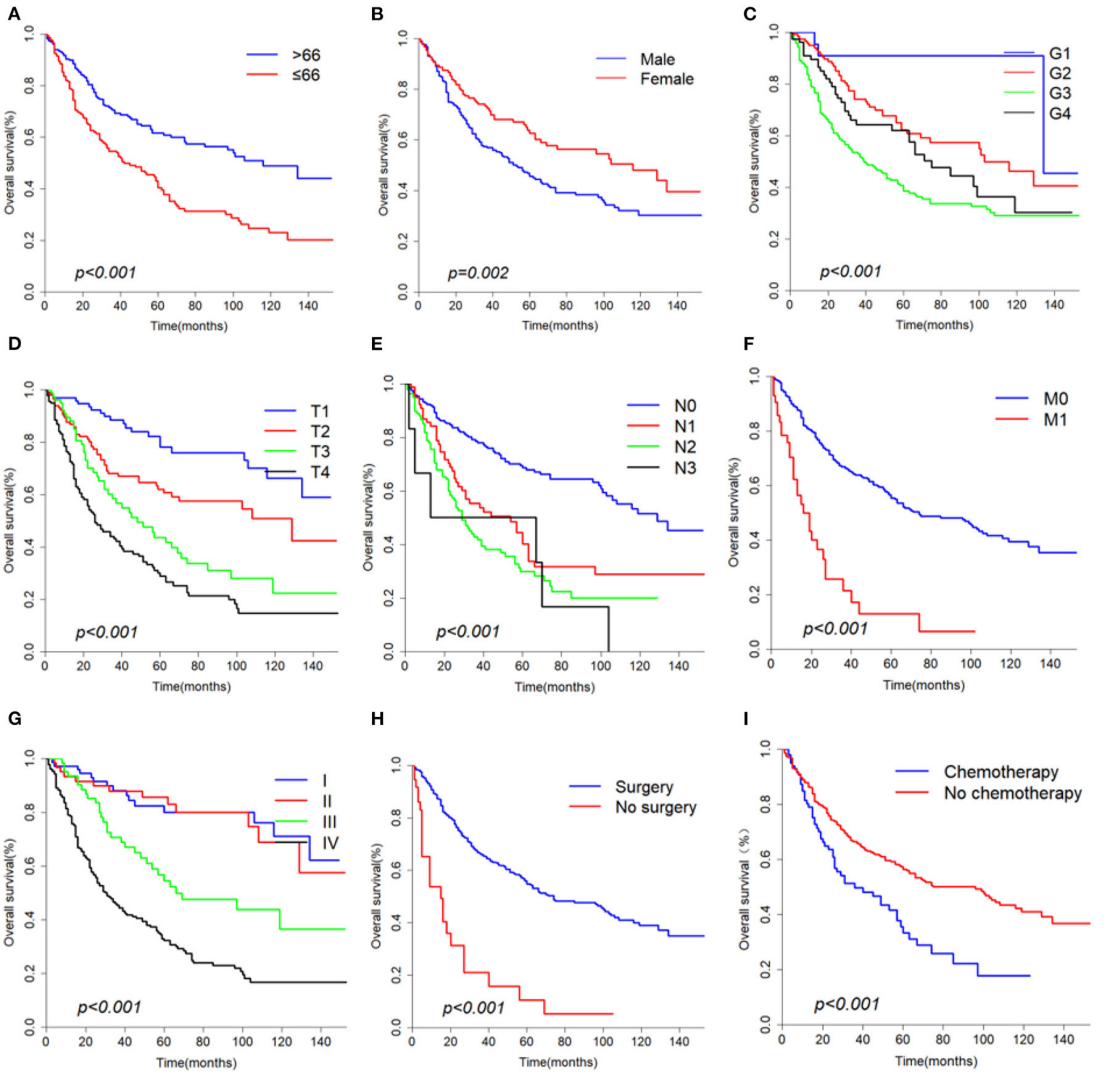
Overall survival curves according to **(A)** Age, **(B)** Gender, **(C)** Grade, **(D)** T classification, **(E)** N classification, **(F)** M classification, **(G)** Stage, **(H)** Surgery, **(I)** Chemotherapy.

The median overall survival of 446 selected patients was 66 months [95% CI (confidence interval): 56–97]. The 3-, 5-, and 10-year OS rates were 63.1, 51.8, and 36.7%, respectively (Figure 2A). The 3-, 5-, and 10-year cancer-specific survival (CSS) rates of patients were 68.2, 60.2, and 48.3%, respectively (Figure 2B). The OS shortened with increasing tumor stage (*p* < 0.001, Figure 3G). Undergoing surgical treatment clearly extended survival time (*p* < 0.001, Figure 3H). The median OS with and without surgery was 74 (95% CI: 60–104) and 15 (95% CI: 9–27) months.

According to univariate analyses (Table 1, Figure 3), nine variables including age, gender, pathologic grade, T classification, N classification, M classification, stage, chemotherapy, and surgery were significant variables related to OS. The results of competing risk analysis and Gray's tests showed that the variables above were still statistically significant for cancer-specific survival (Supplementary Figure 1), except for age (*p* = 0.080). Additionally, age, T classification and stage were identified to be related to other cause-specific death (Supplementary Table 1).

The significant variables were then incorporated into multivariate Cox regression. Variable stage was not included due to its high multicollinearity with T, N, and M classification variables. The final results (Table 2) presented that age (*p* < 0.001), T classification (*p* < 0.001), N classification (*p* < 0.001), M classification (*p* < 0.001), and surgery (*p* < 0.001) were independent risk factors for OS, while sex (*p* = 0.630), pathologic grade (*p* = 0.174) and chemotherapy (*p* = 0.628) were excluded (Table 2). Besides, T classification (*p* < 0.001), N classification (*p* < 0.001), M classification (*p* = 0.002), and surgery (*p* < 0.001) were independent prognostic factors for CSS. Age (*p* < 0.001) was identified as an independent risk factor for other cause-specific death (Supplementary Table 1).

**Table 2:** Multivariate Cox regression analyses of overall survival and cancer-specific survival.

**Characteristics**	**OS**			**CSS**		
	***p-*value**	**HR**	**95% CI**	***p-*value**	**HR**	**95% CI**
Age, ≤ 66 vs. >66	**<0.001**	0.550	0.414–0.730	0.060	0.736	0.534–1.014
Gender, female vs. male	0.630	0.926	0.676–1.268	0.592	1.104	0.770–1.584
Grade	0.174			0.250		
G1	Ref	1		Ref	1	
G2	0.379	1.701	0.521–5.549	0.677	1.478	0.346–6.311
G3	0.093	2.716	0.847–8.712	0.366	2.731	0.656–11.362
G4	0.280	1.933	0.584–6.400	0.577	1.732	0.400–7.490
T classification	**<0.001**			**<0.001**		
T1	Ref	1		Ref	1	
T2	0.056	1.671	0.987–2.828	0.057	1.931	0.981–3.804
T3	<0.001	2.578	1.502 4.425	0.001	3.117	1.573–6.179
T4	<0.001	3.276	1.989–5.394	<0.001	4.282	2.264–8.100
N classification	**<0.001**			**<0.001**		
N0	Ref	1		Ref	1	
N1	0.054	1.464	0.993- 2.158	0.067	1.546	0.970–2.461
N2	<0.001	1.861	1.310–2.645	<0.001	2.459	1.635–3.698
N3	0.013	2.957	1.253–6.979	0.005	3.961	1.520–10.318
M classification, M1 vs. M0	**0.004**	1.990	1.238–3.119	**0.002**	2.172	1.328–3.552
Chemotherapy, No vs. Yes	0.628	1.095	0.760–1.577	0.930	1.018	0.683–1.517
Surgery, No vs. Yes	**<0.001**	2.773	1.720–4.472	**<0.001**	3.078	1.858–5.101

*HR, hazard ratio; CI, confidence interval; Ref, reference*.

*Bold value means the p-value of a statistically significant factor*.

### Propensity Score Matching

**Figure 4:**
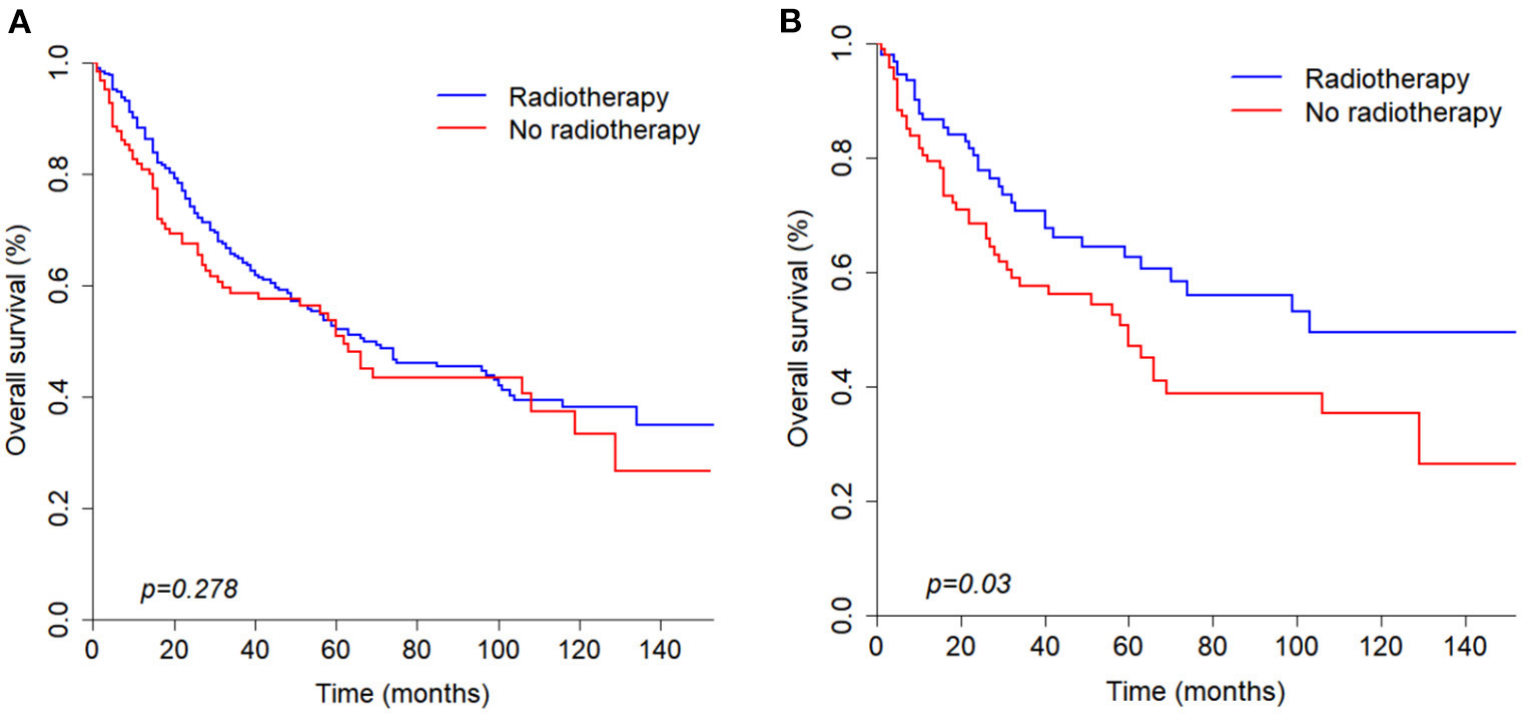
Kaplan-Meier curves of overall survival before **(A)** and after **(B)** propensity score matching analysis based on radiotherapy.

According to the univariate analysis, radiotherapy did not improve the survival of patients (*p* = 0.278). To identify the efficacy of radiotherapy, propensity score matching was performed according to the variables described in the methods. Before PSM, radiotherapy showed a trend toward white race (*p* = 0.037), high grade (*p* = 0.017), large tumor size (*p* = 0.010), positive nodal metastasis (*p* = 0.021), advanced stage (*p* < 0.001) and chemotherapy (*p* = 0.008) (Table 3). After PSM analysis, there were no significant differences in baseline characteristics. In matched groups, radiotherapy significantly improved survival and the median OS was 103 (95% CI: 70–NA) and 60 (95% CI: 32–NA) months (Figure 4).

**Table 3:** Baseline characteristics of patients divided by radiotherapy in the regular and matched groups.

**Characteristics**	**Radiotherapy**	**No radiotherapy**	***p-*value**	**Radiotherapy**	**No radiotherapy**	***p-*value**
	***N* (%)**	***N* (%)**		***N* (%)**	***N* (%)**	
Total	319	127		97	97	
Age (year)			0.831			0.472
>66	151 (47.34)	58 (45.67)		48 (49.48)	54 (55.67)	
≤ 66	168 (52.66)	69 (54.33)		49 (50.52)	43 (44.33)	
Gender			0.136			0.772
Female	105 (32.92)	52 (40.94)		44 (45.36)	41 (42.27)	
Male	214 (67.08)	75 (59.06)		53 (54.64)	56 (57.73)	
Ethnicity			0.037			0.069
White	270 (84.64)	101 (79.53)		78 (80.41)	83 (85.57)	
Black	33 (10.34)	11 (8.66)		15 (15.46)	6 (6.19)	
Other	16 (5.02)	15 (11.81)		4 (4.12)	8 (8.25)	
Laterality			0.418			0.773
Left	138 (43.26)	61 (48.03)		42 (43.30)	45 (46.39)	
Right	181 (56.74)	66 (51.97)		55 (56.70)	52 (53.61)	
Marital status			0.742			1.000
Divorced/other	116 (36.36)	49 (38.58)		34 (35.05)	34 (35.05)	
Married	203 (63.64)	78 (61.42)		63 (64.95)	63 (64.95)	
Grade			0.017			0.447
G1	12 (3.76)	13 (10.24)		6 (6.19)	7 (7.22)	
G2	82 (25.71)	40 (31.50)		36 (37.11)	28 (28.87)	
G3	168 (52.66)	53 (41.73)		43 (44.33)	43 (44.33)	
G4	57 (17.87)	21 (16.54)		12 (12.37)	19 (19.59)	
T stage			0.010			0.317
T1	62 (19.44)	38 (29.92)		25 (25.77)	29 (29.90)	
T2	79 (24.76)	40 (31.50)		35 (36.08)	25 (25.77)	
T3	68 (21.32)	20 (15.75)		19 (19.59)	17 (17.53)	
T4	110 (34.48)	29 (22.83)		18 (18.56)	26 (26.80)	
N stage			0.021			0.748
N0	143 (44.83)	76 (59.84)		54 (55.67)	55 (56.70)	
N1	65 (20.38)	14 (11.02)		16 (16.49)	11 (11.34)	
N2	106 (33.23)	36 (28.35)		26 (26.80)	30 (30.93)	
N3	5 (1.57)	1 (0.79)		1 (1.03)	1 (1.03)	
M stage			0.062			0.479
M0	294 (92.16)	109 (85.83)		89 (91.75)	85 (87.63)	
M1	25 (7.84)	18 (14.17)		8 (8.25)	12 (12.37)	
Stage			<0.001			0.180
I	44 (13.79)	33 (25.98)		21 (21.65)	25 (25.77)	
II	36 (11.29)	26 (20.47)		20 (20.62)	15 (15.46)	
III	55 (17.24)	14 (11.02)		20 (20.62)	11 (11.34)	
IV	184 (57.68)	54 (42.52)		36 (37.11)	46 (47.42)	
Chemotherapy			0.008			1.000
Yes	84 (26.33)	18 (14.17)		14 (14.43)	15 (15.46)	
No	235 (73.67)	109 (85.83)		83 (85.57)	82 (84.54)	
Surgery			0.260			0.781
Yes	296 (92.79)	113 (88.98)		91 (93.81)	89 (91.75)	
No	23 (7.21)	14 (11.02)		6 (6.19)	8 (8.25)	

### Establishment and Validation of the Nomogram

**Figure 5:**
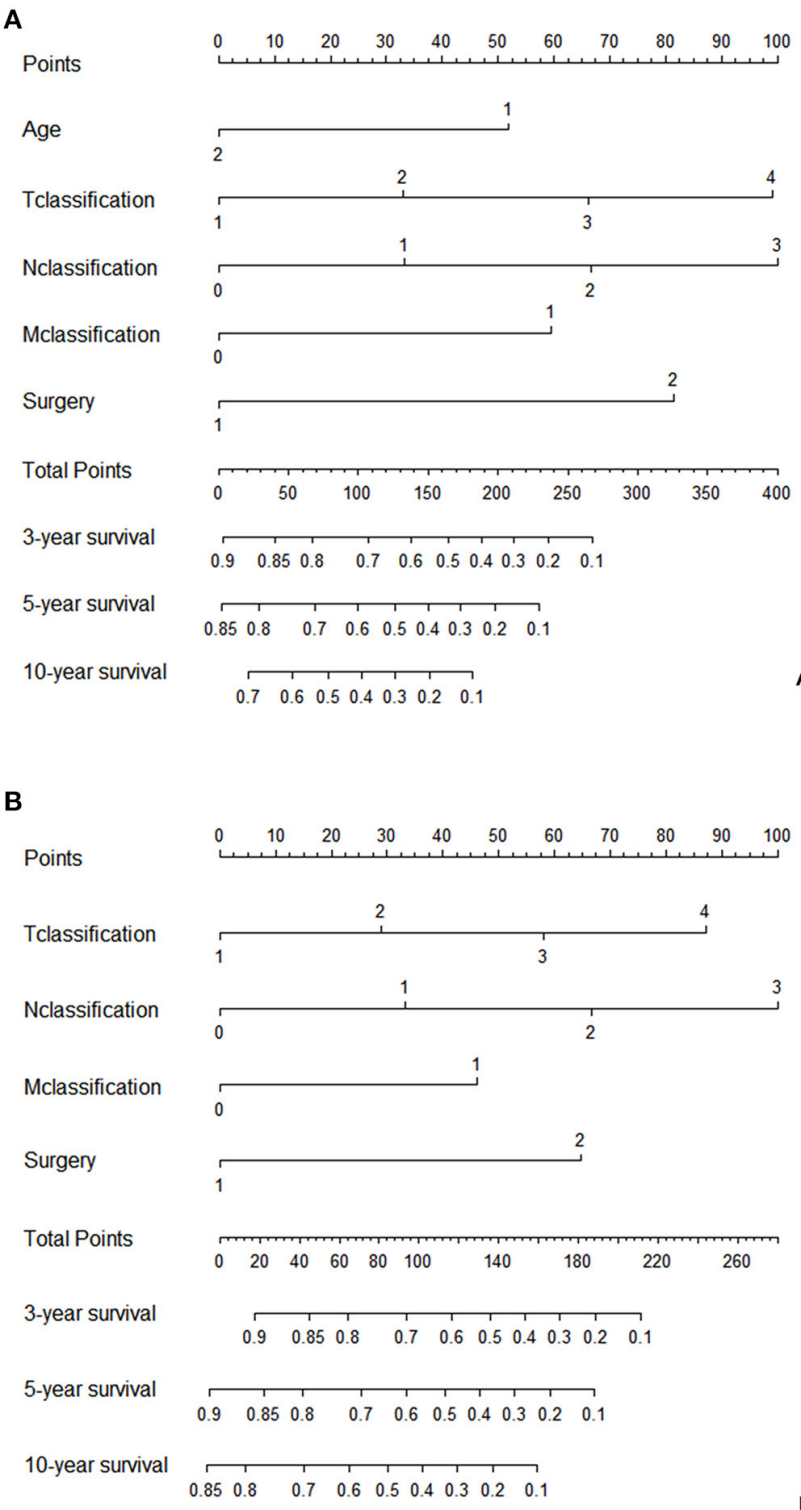
Nomograms for prediction of 3-, 5-, and 10-year **(A)** OS rates; **(B)** CSS rates.

The significant variables based on the final multivariate models were included to create the nomograms for OS and CSS. Figure 5 presented two nomograms which predict 3-, 5-, and 10-year OS and CSS. The C-indexes of the nomograms for OS and CSS were 0.747 and 0.780, which indicated good discrimination of the two nomograms. The calibration curves also showed that the predicted survival probability matched well with the observed survival probability at the 3-, 5-, and 10-year time points (Supplementary Figure 2).

**Figure 6:**
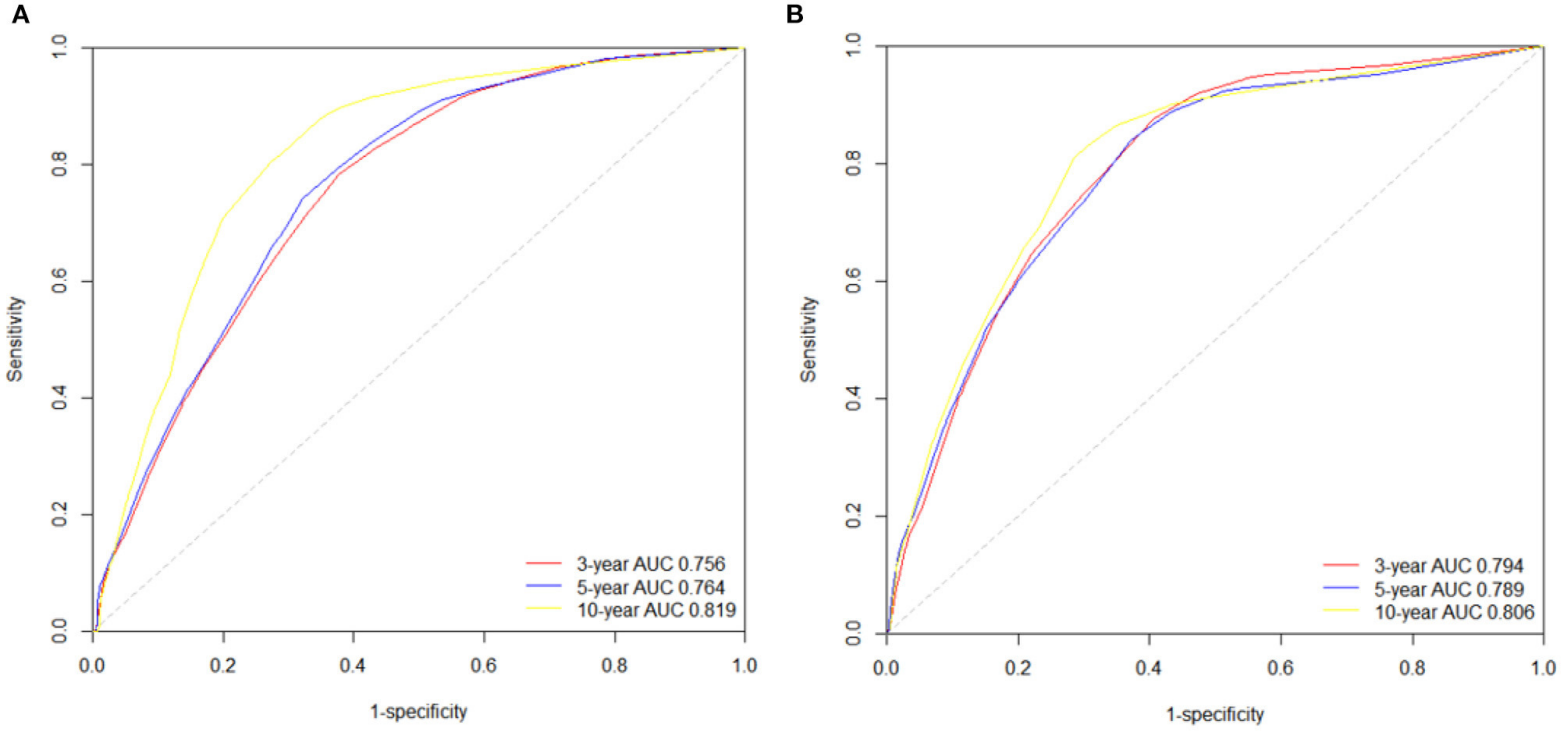
The time-dependent ROC curves of the nomograms predicting 3-,5-, and 10-year **(A)** OS rates; **(B)** CSS rates.

We also used time-dependent receiver operating characteristic (ROC) curves and the area under the ROC curve (AUC) to validate the efficacy of nomograms. As shown in Figure 6, the area under the curve (AUC) values of ROC were 0.756, 0.764 and 0.819 regarding for nomogram predicting 3-, 5- and 10- year OS, respectively. Likewise, the 3-, 5-, and 10-year AUC values of nomogram predicting for CSS were 0.794, 0.789 and 0.806, respectively.

## Discussion

In this study, we analyzed data from a large cohort of 446 patients with parotid gland adenocarcinoma not otherwise specified and constructed two nomograms. Our findings may present a comprehensive viewpoint on the characteristics and prognosis of PANOS patients.

Our study found that some clinical features of PANOS were in accordance with previously published reports, including a male predominance, a tendency to lymph node invasion, a propensity for high-grade tumors and more patients who were diagnosed with advanced stage III/IV disease (8, 14, 15).

With respect to survival, the 5 and 10-year overall survival rates of PANOS patients were 51.8 and 36.7%, which were significantly lower than those of mucoepidermoid carcinoma and adenoid cystic carcinoma (4–6). According to similar SEER analyses, Sun et al. (4) showed that the 5 and 10-year OS rates of mucoepidermoid carcinoma were 83.2 and 73.6%, and the 5-year OS rates of adenoid cystic carcinoma in Tasoulas's study (6) were 81%. These findings indicate that PANOS has a relatively poorer prognosis among major parotid gland carcinoma subtypes, which calls for our attention.

Surgical resection is still the principal treatment for parotid gland carcinomas which has considerable effects on patient survival (16–18). In this study for PANOS patients, surgery notably prolonged OS among patients with PANOS (*p* < 0.001). The median overall survival of patients who underwent surgery increased by nearly 5 years compared to those who did not. Multivariate Cox analyses also revealed that surgery was an independent favorable prognostic factor both for OS and CSS.

Many studies have shown that radiotherapy can prolong survival of parotid gland carcinoma patients (19, 20). In our study, the characteristics between radiotherapy and non-radiotherapy patients were imbalanced before propensity score matching analysis and univariate analysis showed that radiotherapy was not significantly correlated with OS (*p* = 0.278). After PSM analysis was conducted, the final results showed that radiotherapy can significantly improved survival (*p* = 0.03). Therefore, radiotherapy still plays a significant role in the survival of PANOS patients.

Previous studies have shown that age is an important prognostic factor for survival in patients with PANOS (8, 21), and our study also found that younger patients had a better prognosis. In our study, age was an independent prognostic factor of OS and other cause-specific death, but it had no effect on CSS. This may be explained by the fact that PANOS has a relatively longer survival time compared with other cancers (3), and that older patients may suffer from death caused by competing events like cardiovascular events, which may hinder the occurrence of cancer-specific death.

Unsurprisingly, T, N, and M classifications were independent risk factors both for OS and CSS. A larger range of tumor extension, more involved lymph nodes, or distant metastasis were correlated with shortened survival time.

It is well known that the American Joint Committee on Cancer (AJCC) Staging is a commonly used prognostic tool for malignancies (22). Nevertheless, this staging system is not specially designed for individuals and did not include important prognostic factors such as age, tumor differentiation and the treatments that patients have received. The nomogram includes a variety of cancer-related risk factors and can individually predict the survival rate of patients in a visual way (23, 24). Therefore, we established and validated two nomograms to predict the 3-, 5-, and 10-year survival rates in a quantitive way, which may assist physicians in evaluating patient's prognoses for PANOS.

This study has certain limitations. Firstly, in the process of patient selection, many patients were excluded due to the absence of information such as pathological grade and stage, which may have led to the extraction of inprecise OS and CSS for the patients. Secondly, the lack of data on chemotherapy regimens and clinical symptoms, such as pain or tenderness may limit the ability to identify their influences on the prognosis of PANOS patients. Thirdly, the pathological classification of parotid carcinoma is a challenge because its subtypes are so diverse and we cannot examine the original clinicopathological information, which may lead to biased information.

## Conclusions

In summary, a population-based method was used to present the clinicopathological features and prognosis of patients with PANOS based on data extracted from the SEER database. We demonstrated that age, T, N and M classifications and surgery were independent prognostic factors for OS; T, N and M classifications and surgery were independent risk factors for CSS. In addition, age was independently correlated with other cause-specific death. Moreover, we developed two nomograms predicting the 3-, 5-, and 10-year OS and CSS in patients with PANOS, which can visually and effectively evaluate the prognosis of individuals.

## Data Availability Statement

The original contributions presented in the study are included in the article/Supplementary Materials, further inquiries can be directed to the corresponding author/s.

## Ethics Statement

Ethical review and approval was not required for the study on human participants in accordance with the local legislation and institutional requirements. Written informed consent for participation was not required for this study in accordance with the national legislation and the institutional requirements.

## Author Contributions

Z-MW and Z-LX conceived the idea of the study. Z-MW conducted data extraction and analysis, and wrote the manuscript. All authors discussed the results and revised the manuscript.

## Funding

This work was supported by the National Natural Science Foundation of China (Grant No. 81960525), Science and the Technology innovation project of Shanghai (Grant No. 19DZ1930903), Pudong New Area Science and Technology Development Fund (Grant No. PKJ2018-Y02) and Natural Science Foundation of Jiangxi (Grant No. 20192BAB205071).

## Conflict of Interest

The authors declare that the research was conducted in the absence of any commercial or financial relationships that could be construed as a potential conflict of interest.

## Publisher's Note

All claims expressed in this article are solely those of the authors and do not necessarily represent those of their affiliated organizations, or those of the publisher, the editors and the reviewers. Any product that may be evaluated in this article, or claim that may be made by its manufacturer, is not guaranteed or endorsed by the publisher.

## Supplementary Material

Supplementary Figure 1 Competing risk analyses according to **(A)** Age, **(B)** Gender, **(C)** Grade, **(D)** T classification, **(E)** N classification, **(F)** M classification, **(G)** Stage, **(H)** Surgery, **(I)** Chemotherapy. CSD, cancer-specific deaths; OD, other deaths.

Supplementary Figure 2 Calibration curves of the nomograms predicting 3-,5- and 10-year **(A)** OS rates; **(B)** CSS rates.

Supplementary Table 1 Multiple cox regression of other cause-specific death. HR, hazard ratio; CI, confidence interval; Ref, reference.
