# Corrigendum: Establishment and validation of prognostic nomograms for patients with parotid gland adenocarcinoma not otherwise specified: a SEER analysis from 2004 to 2016

**DOI:** 10.3389/fsurg.2023.1341579

**Published:** 2023-12-22

**Authors:** Zi-Meng Wang, Zuo-Lin Xiang

**Affiliations:** Department of Radiation Oncology, Shanghai East Hospital, School of Medicine, Tongji University, Shanghai, China

**Keywords:** parotid gland, adenocarcinoma not otherwise specified, SEER database, prognosis, nomogram

A Corrigendum on Establishment and validation of prognostic nomograms for patients with parotid gland adenocarcinoma not otherwise specified: a SEER analysis from 2004 to 2016 By Wang ZM, Xiang ZL (2022) Front Surg. 8:799452. doi: 10.3389/fsurg.2021.799452

**Incorrect Funding**

In the published article, there was an error in the Funding statement. The grant number currently reads as “Science and Technology Commission of Shanghai Municipality (Grant No. 19DZ1930900)”. However, it should be corrected to “Science and Technology Commission of Shanghai Municipality (Grant No. 19DZ1930903)”.

The correct Funding statement appears below.

