# Supplementary figures and images for: Establishment and Validation of Prognostic Nomograms for Patients With Parotid Gland Adenocarcinoma Not Otherwise Specified: A SEER Analysis From 2004 to 2016
Source: Front Surg. 2022 Jan 11;8:799452. doi: 10.3389/fsurg.2021.799452 (PMC8786720; doi:10.3389/fsurg.2021.799452)

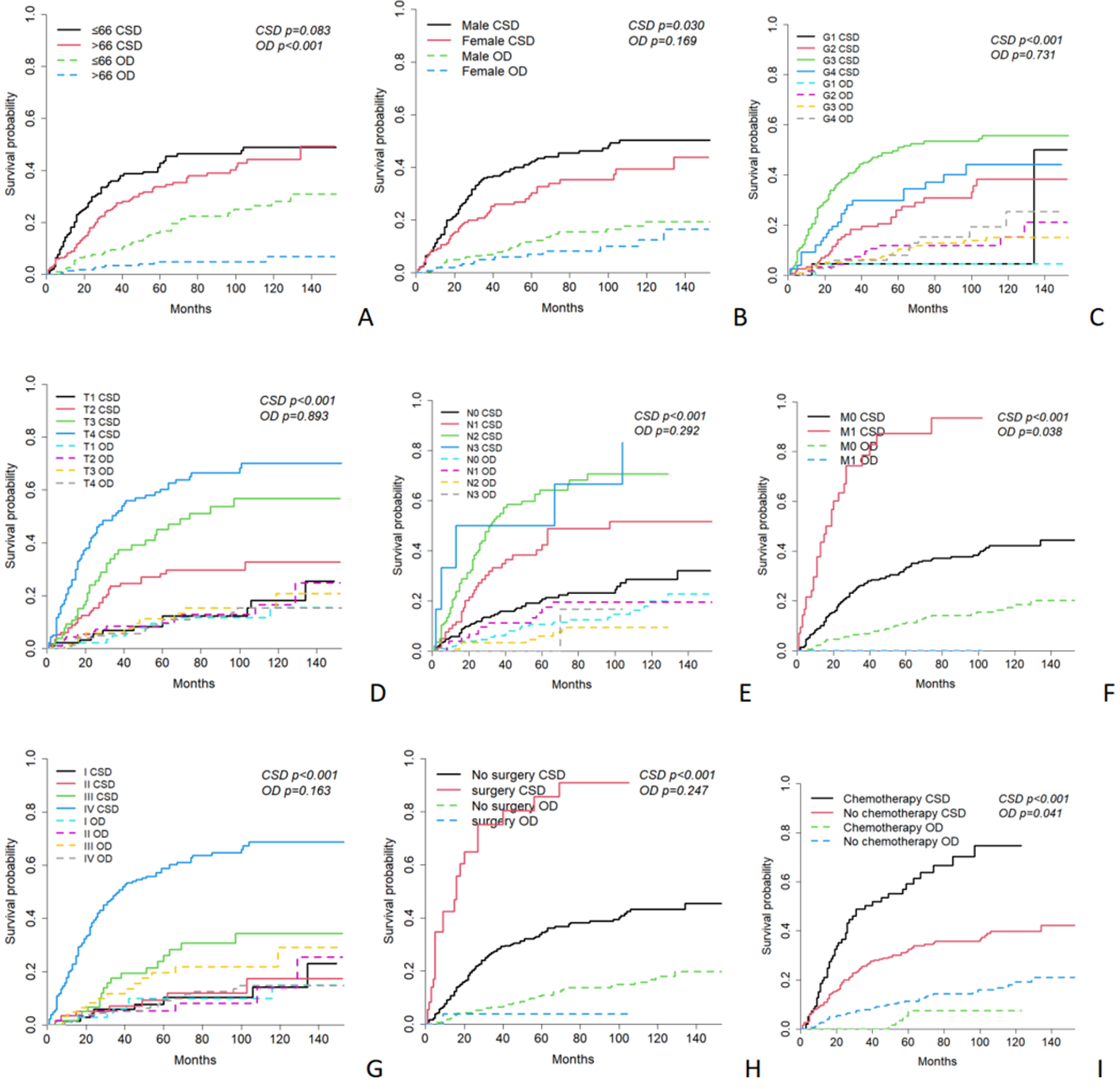

Supplement: Supplementary Figure 1 — Competing risk analyses according to (A) Age, (B) Gender, (C) Grade, (D) T classification, (E) N classification, (F) M classification, (G) Stage, (H) Surgery, (I) Chemotherapy. CSD, cancer-specific deaths; OD, other deaths. [file Image_1.tif]

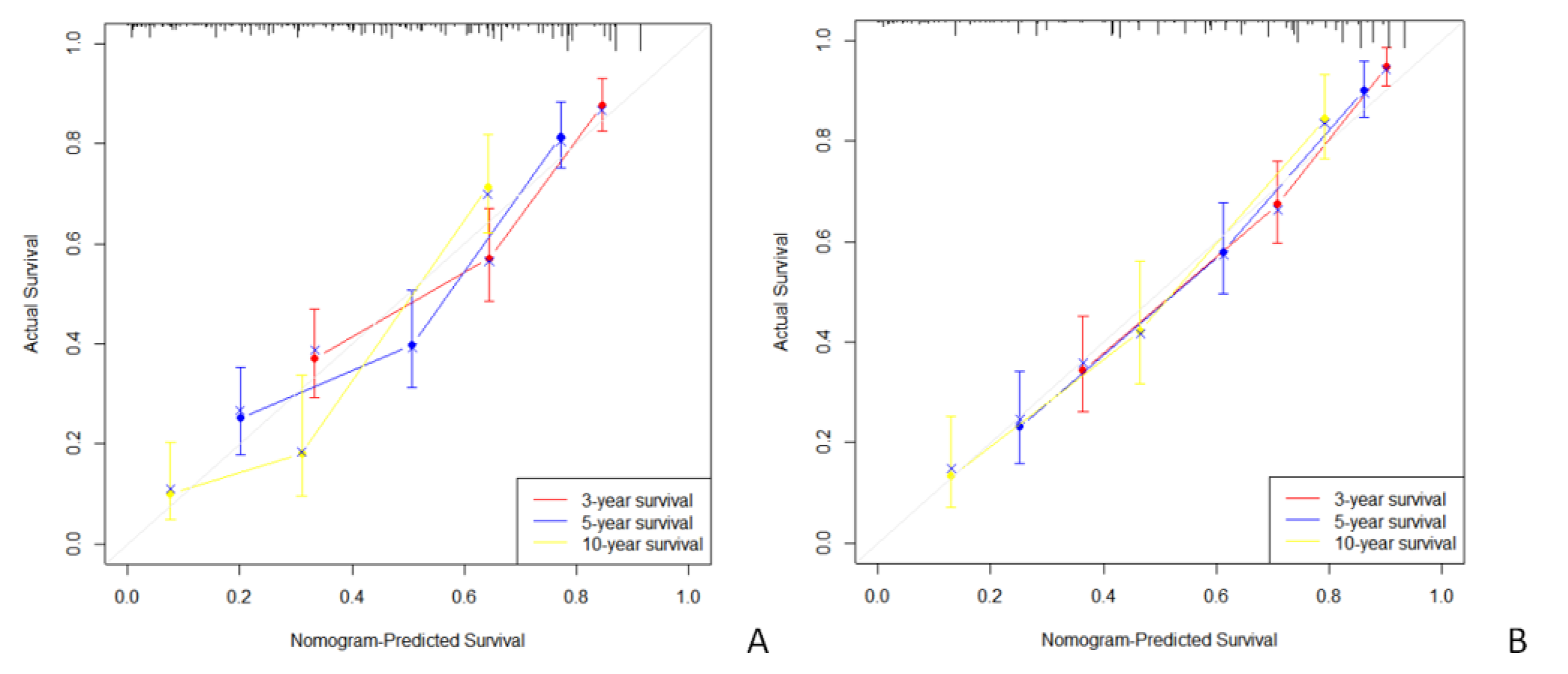

Supplement: Supplementary Figure 2 — Calibration curves of the nomograms predicting 3-,5- and 10-year (A) OS rates; (B) CSS rates. [file Image_2.tif]
