# Supplementary material for: Establishment and Validation of Prognostic Nomograms for Patients With Parotid Gland Adenocarcinoma Not Otherwise Specified: A SEER Analysis From 2004 to 2016
Source: Front Surg. 2022 Jan 11;8:799452. doi: 10.3389/fsurg.2021.799452 (PMC8786720; doi:10.3389/fsurg.2021.799452)
Supplement: Supplementary Table 1 — Multiple cox regression of other cause-specific death. HR, hazard ratio; CI, confidence interval; Ref, reference. [file Table_1.DOCX]

Table S1 Multiple Cox regression of other cause-specific death.

| Characteristics | *p* value | HR | 95% CI |
| --- | --- | --- | --- |
| Age, ≤66 vs. >66 | **<0.001** | 0.177 | 0.088-0.358 |
| T classification | 0.148 | 1.202 | 0.937-1.544 |
| T1 | Ref | 1 |  |
| T2 | 0.575 | 1.272 | 0.548-2.950 |
| T3 | 0.138 | 1.986 | 0.803-4.913 |
| T4 | 0.214 | 1.698 | 0.736-3.918 |

HR, hazard ratio; CI, confidence interval.Ref:reference.
